# Commuting and health in Cambridge: a study of a 'natural experiment' in the provision of new transport infrastructure

**DOI:** 10.1186/1471-2458-10-703

**Published:** 2010-11-16

**Authors:** David Ogilvie, Simon Griffin, Andy Jones, Roger Mackett, Cornelia Guell, Jenna Panter, Natalia Jones, Simon Cohn, Lin Yang, Cheryl Chapman

**Affiliations:** 1 Medical Research Council Epidemiology Unit and UKCRC Centre for Diet and Activity Research (CEDAR), Institute of Public Health, Cambridge, UK; 2 School of Environmental Sciences, University of East Anglia and UKCRC Centre for Diet and Activity Research (CEDAR), Norwich, UK; 3 Centre for Transport Studies, University College London, London, UK; 4 General Practice and Primary Care Research Unit, Institute of Public Health, Cambridge, UK

## Abstract

**Background:**

Modifying transport infrastructure to support active travel (walking and cycling) could help to increase population levels of physical activity. However, there is limited evidence for the effects of interventions in this field, and to the best of our knowledge no study has convincingly demonstrated an increase in physical activity directly attributable to this type of intervention. We have therefore taken the opportunity presented by a 'natural experiment' in Cambridgeshire, UK to establish a quasi-experimental study of the effects of a major transport infrastructural intervention on travel behaviour, physical activity and related wider health impacts.

**Design and methods:**

The *Commuting and Health in Cambridge *study comprises three main elements: a cohort study of adults who travel to work in Cambridge, using repeated postal questionnaires and basic objective measurement of physical activity using accelerometers; in-depth quantitative studies of physical activity energy expenditure, travel and movement patterns and estimated carbon emissions using household travel diaries, combined heart rate and movement sensors and global positioning system (GPS) receivers; and a longitudinal qualitative interview study to elucidate participants' attitudes, experiences and practices and to understand how environmental and social factors interact to influence travel behaviour, for whom and in what circumstances. The impacts of a specific intervention - the opening of the Cambridgeshire Guided Busway - and of other changes in the physical environment will be examined using a controlled quasi-experimental design within the overall cohort dataset.

**Discussion:**

Addressing the unresolved research and policy questions in this area is not straightforward. The challenges include those of effectively combining different disciplinary perspectives on the research problems, developing common methodological ground in measurement and evaluation, implementing robust quantitative measurement of travel and physical activity behaviour in an unpredictable 'natural experiment' setting, defining exposure to the intervention, defining controls, and conceptualising an appropriate longitudinal analytical strategy.

## Background

A low level of physical activity increases the risk of obesity and many preventable chronic diseases including coronary heart disease, type 2 diabetes and cancer of the colon [1]. Observational studies suggest that two and half hours of moderate-intensity physical activity per week is enough to provide substantial health benefits [2], but most adults in countries such as the UK do not achieve this [1,3]. Increasing the population level of physical activity, particularly among the most sedentary, has been described as the 'best buy' for improving public health [4] and is an established priority of health policy [5].

Efforts to promote physical activity have traditionally been directed at 'high-risk' individuals and focused on promoting sport, recreation, or health-directed exercise [6]. However, there is limited evidence that such approaches are effective in increasing and maintaining levels of physical activity in the medium-to-long term [7] and no definitive evidence of any upward trend in overall physical activity from surveillance data for England [8]. Greater public health benefits may therefore accrue from a population strategy to shift the distribution of physical activity in the population as a whole than from further efforts to target high-risk groups [9]. This is likely to require a combination of interventions at multiple levels [10] to address different domains of physical activity (domestic, occupational, recreational and transport) and the behaviour- and context-specific determinants of different forms of activity such as walking or cycling [11,12].

One reason for the limited success of previous interventions may be that people's capacity to respond to public health advice to take more exercise is limited by their surroundings and the opportunities open to them. Programme guidance on physical activity and the environment issued by the National Institute for Health and Clinical Excellence (NICE) has drawn attention to the lack of studies examining whether changing the physical environment leads to changes in physical activity. The guidance specifically identified a need for more, and more rigorous, studies of this kind involving longitudinal designs, comparisons with control groups, and robust measures of physical activity [13]. These features are important to allow the effectiveness, cost-effectiveness and distributional impacts of this type of intervention to be meaningfully compared with those of other approaches to promoting physical activity, for example through consultations in primary care. The lack of credible evidence from intervention studies reflects the challenges of researching the effects of this type of intervention, and public health researchers are increasingly attempting to address this lack of evidence by studying the effects of 'natural experiments' [14-16]. These include situations in which the environment is modified, as in the M74 study of urban motorway construction in Glasgow [17,18] or the iConnect study of walking and cycling infrastructure projects around the UK [19], and others in which people move to a completely new environment, as in the RESIDE study in Western Australia [20].

Modifying transport infrastructure to support active travel (walking and cycling), for example by constructing cycle routes or redesigning roads to discourage car use, is one way of modifying the physical environment that was identified in the recent Foresight report as one of the top five recommendations for tackling obesity in the UK [21]. The considerable potential for people to incorporate walking or cycling into their daily routines makes this an attractive strategy for increasing population levels of physical activity. However, several reviews have highlighted the limited quantity and quality of existing studies of the effects of this type of intervention, the very limited evidence that such interventions have been effective in promoting physical activity, and the missed opportunities for rigorous health-oriented studies of the effects of recent major innovations such as the impact of congestion charging in London on physical activity [13,22-26]. It cannot be assumed that people who take up more active travel will become more physically active overall, because the increase in energy expenditure while travelling may be counterbalanced - or even outweighed - by a compensatory decrease in leisure-time physical activity [14]. There is a particular lack of evidence on the relationships between public transport and active travel and on the effects of interventions to improve public transport infrastructure, which may be especially important in a country such as the UK where many people live too far from their workplace to walk or cycle the entire journey. Several studies have shown that using public transport can involve a substantial daily quantity of walking and that commuters who use public transport tend to walk more than those who travel by car [27-30]. One study has also reported a correspondence between an increase in cycling and a positive shift in the distribution of overall physical activity in the targeted local populations following a multifaceted intervention including some infrastructural changes [31]. To the best of our knowledge, however, no study has yet convincingly demonstrated that investing in new transport infrastructure has led to an increase in physical activity in the local population directly attributable to the intervention [13,26].

We have therefore taken the opportunity presented by a 'natural experiment' in Cambridgeshire, UK to establish a quasi-experimental study of the effects of a major transport infrastructural intervention on travel behaviour, physical activity and related wider health impacts. This study is designed to contribute new evidence relating to several unresolved research and policy questions in this area, including (a) the effectiveness of interventions in promoting a shift from car use towards more sustainable modes of transport; (b) whether walking and cycling can be promoted as part of longer public transport journeys by improving public transport provision; (c) the contribution of active travel to overall physical activity; and (d) the wider health impacts of changes in travel behaviour.

### Aims and objectives

The aim of the study is to address the following primary research question:

1. Is investment in new high-quality transport infrastructure associated with an increase in the use of active modes of travel (walking and cycling)? 

and the following secondary research questions:

2. What are the wider health impacts of changes in travel behaviour in terms of overall physical activity, wellbeing, sickness absence and carbon emissions?

3. What are the determinants of the use and uptake of active modes of travel?

4. How are any changes in travel behaviour distributed in the population?

5. How are travel behaviour and changes in travel behaviour embedded in and shaped by the wider social context?

6. Are changes in travel behaviour sustained over time?

in a quasi-experimental cohort study of adults who travel to work in Cambridge, combined with nested in-depth quantitative and qualitative studies.

### Design and methods

#### Overall research design

The *Commuting and Health in Cambridge *study comprises three main elements:

1. A cohort study of travel behaviour, physical activity and wider health impacts in adults who travel to work in Cambridge, using repeated postal questionnaires and basic objective measurement of physical activity using accelerometers

2. In-depth quantitative studies of subgroups of participants to investigate physical activity energy expenditure, travel and movement patterns and estimated carbon emissions using household travel diaries, combined heart rate and movement sensors and global positioning system (GPS) receivers

3. A longitudinal qualitative interview study to elucidate participants' attitudes, experiences and practices and to understand how environmental and social factors interact to influence travel behaviour, for whom and in what circumstances.

The impacts of a specific intervention - the opening of the Cambridgeshire Guided Busway (defined below under 'Intervention') - and of other changes in the physical environment will be examined using a controlled quasi-experimental design within the overall cohort dataset.

### Setting

**Figure 1:**
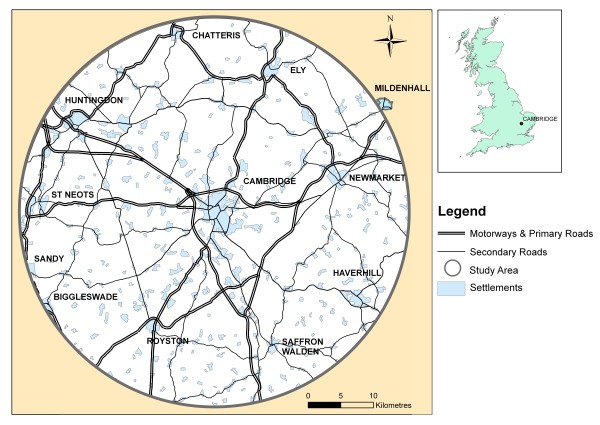
**Study area**. © Crown Copyright 2009. An Ordnance Survey/EDINA supplied service

The city of Cambridge lies approximately 80 km northeast of London and has a population of approximately 108,000 [32]. The Cambridge sub-region includes the smaller cathedral city of Ely and market towns such as Huntingdon, Newmarket, Royston and St Neots as well as a large number of small settlements (Figure 1). Unlike most cities in the UK, Cambridge has a distinct cycling culture related to a combination of factors including its flat topography, its large student population and the traffic congestion in its historic city centre. This is reflected in the 2008 Cambridgeshire Travel for Work survey, which found that 21.1% of those surveyed cycled to work [33] - substantially higher than the UK average of 3.3% [32]. Conversely, the proportion of employees who travel to work by car is lower in Cambridge (54.9%) than in the UK as a whole (61.5%) [32]. Cambridge has good road transport links: primary roads connect it to surrounding towns, the M11 motorway (freeway) links it with London and the south, and the A14 trunk road provides a major east-west route. The city is also well served by frequent train services to London and some other more distant cities, as well as to certain towns and villages in its immediate hinterland. On the other hand, the cost of housing in the city of Cambridge is high by UK standards and the Cambridge sub-region is a predominantly rural area with limited rural public transport and a relatively high level of rural car ownership [32,34].

### Participants

#### Inclusion criteria

The study population comprises adults aged 16 and over who work in areas of Cambridge served by the Cambridgeshire Guided Busway (CGB) and live within a radius of approximately 30 kilometres of the city centre (Figure 1) but not within the same immediate area of the city as their workplace. Within this definition, participants are eligible for inclusion irrespective of their employer, workplace, type or grade of occupation, length of employment contract or working hours; whether they also work at other locations; and whether they have any disability that may limit their mobility. Participants are ineligible if they are currently taking part in another research study that involves measuring their physical activity, or if they live in on-site staff accommodation associated with their workplace and therefore do not routinely commute to the site.

#### Recruitment

Since the study is focused on travel to work, participants are recruited through workplaces rather than from a general population sampling frame such as an electoral or primary care register. However, in the interests of data protection and to assure participants of the independence of the study from their employers, participants are not recruited through corporate staff databases. Instead, they are approached using a combination of recruitment stands, newspaper and magazine advertisements, posters, fliers, and announcements distributed on the investigators' behalf by employers through corporate email distribution lists, intranets and staff newsletters. Participants who opt in to the study by responding to any of these recruitment methods are entered into a prize draw to win one of eight £50 gift vouchers.

Recruitment commenced in March 2009. The majority of participants were recruited in 2009 and took part in baseline data collection between May and October 2009, with limited additional recruitment to the cohort and baseline data collection from the new participants in the corresponding period of 2010. Written informed consent was obtained from all participants.

### Intervention

**Figure 2:**
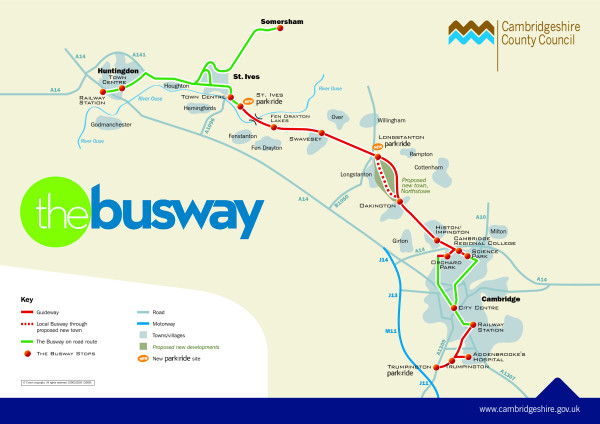
**Guided busway route**. Credit: Cambridgeshire County Council. Reproduced with permission

**Figure 3:**
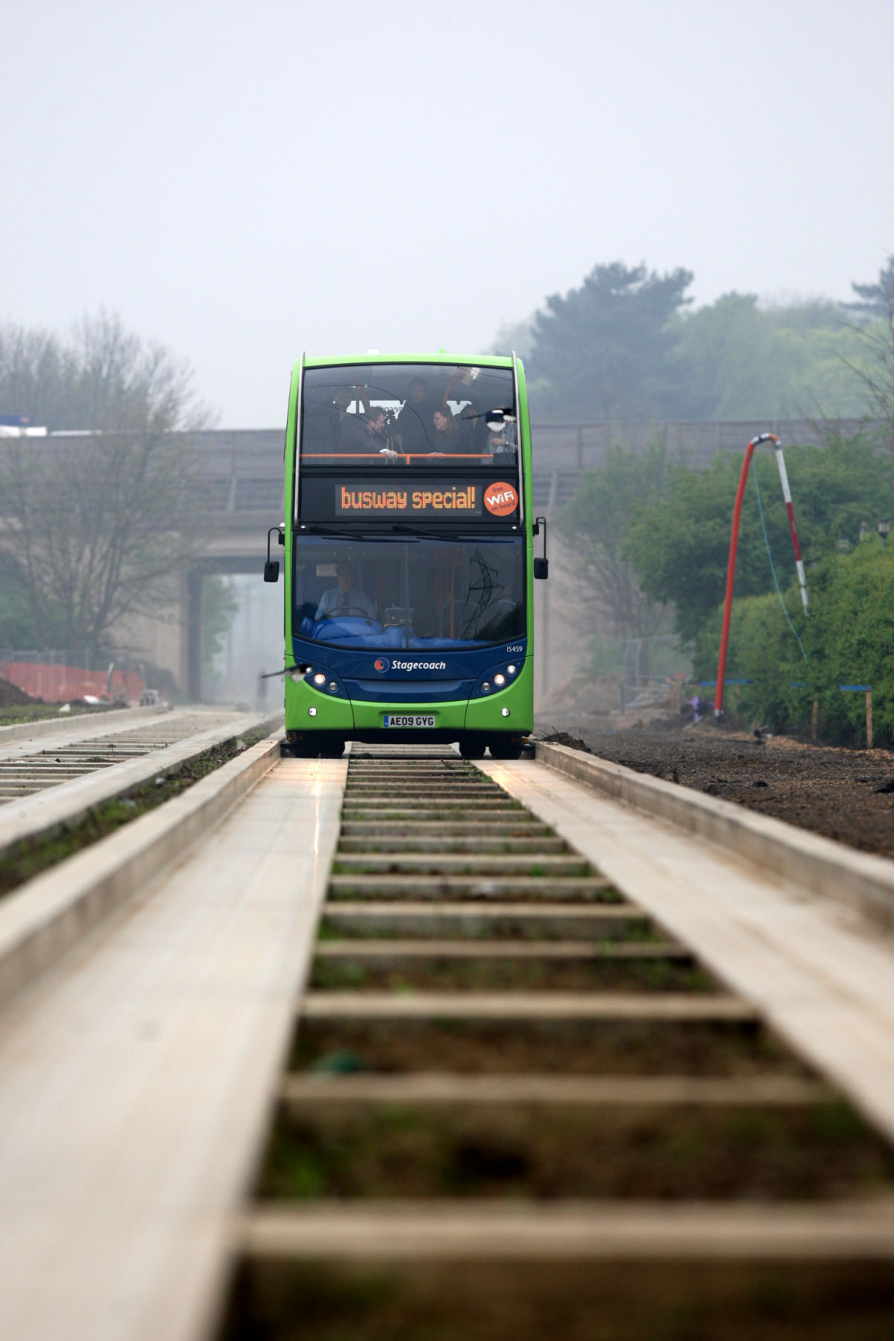
**Bus on guided busway**. Credit: Cambridgeshire County Council. Reproduced with permission

When the Cambridgeshire Guided Busway opens, it will be the longest of its kind in the world [35]. This major new piece of transport infrastructure will link towns and villages to the northwest of Cambridge with the Cambridge Science Park, the city centre and the Cambridge Biomedical Campus at the Addenbrooke's Hospital site on the southern fringe of the city, a site that is said to generate more traffic than any other in the East of England. Buses will run on a completely segregated track along most of the route, avoiding traffic congestion both on the A14 trunk road approaching Cambridge and between the railway station and the hospital (Figure 2, Figure 3). A new high-quality bidirectional off-road cycle route is also to be provided adjacent to the busway. The busway is central to the plans for the new town of Northstowe, which is planned to be built on a former military site adjacent to the route northwest of Cambridge.

Like most infrastructural developments of this kind, the construction of the busway is not taking place in isolation and it cannot easily be regarded as a discrete intervention for the purposes of evaluation. Concurrent changes in the local built environment include a new housing development at Orchard Park adjacent to the route on the northwestern edge of the city [36]; the construction of a new access road and cycle route to the Cambridge Biomedical Campus from the southwest [37]; and other new infrastructure for cycling in and around Cambridge funded by the national Cycling Towns programme [38]. Furthermore, the completion and opening of the busway has been repeatedly delayed. Its implementation is therefore best regarded as a phased process. New buses have been operating on the road network, and parts of the cycle route have been opened in stages, the majority of the northern section having been open for use by pedestrians and cyclists since October 2009. The full opening of the busway is now not expected to take place until early 2011, nearly two years later than originally announced [35].

### Comparisons

The likely catchment area (or zone of influence) of new transport infrastructure of this kind is also subject to considerable uncertainty. It is likely to differ between urban and rural parts of the route, because rural commuters may be more likely to use feeder modes of transport such as park-and-ride and cycling to access the new public transport services. Furthermore, it does not necessarily follow that 'exposure' to the intervention is limited to (or greatest among) residents of neighbourhoods or settlements immediately adjacent to the route, since commuters from other areas could intersect with busway services at transport interchanges such as Cambridge railway station. Defining 'intervention' and 'control' groups is therefore not as straightforward as it might be in the context of a typical parallel-group controlled intervention study.

On recruitment to the study, members of the cohort are allocated to provisional 'intervention', 'control' and 'reserve' groups on the basis of their home postcodes. An *a priori *'intervention' area has been defined comprising a set of unit postcodes (the smallest unit of postal geography in the UK) that fall within, or encroach upon, either (a) a 600 metre road network distance buffer around the stops along the urban sections of the route or (b) larger areas encompassing the towns and villages along the rural sections of the route. The urban buffer reflects the pedestrian access distance used to model the transport impacts of the busway at the planning stage, while the rural buffers are intended to reflect the potential use of other feeder modes including the car. An *a priori *'control' area has also been defined: this comprises two areas of Cambridge city (Barnwell and Romsey Town) with similar aggregate socioeconomic and spatial characteristics to those of the urban parts of the 'intervention' area but with no direct access to the busway, as well as a 180° sector of the rural portion of the study area on the opposite side of the city to the northwestern radial axis of the main section of the busway. All members of the cohort living within the study area but not within the 'intervention' and 'control' areas are assigned to the 'reserve' group.

The influence of the busway will not, in practice, be limited by any arbitrarily-selected distance buffer. The assumptions implicit in defining the boundaries of the 'intervention' and 'control' areas will subsequently be tested in two ways. First, the network distance from home to the nearest guided busway stop or access point will be entered as a covariate in analysis of the determinants of changes in travel behaviour in the cohort. Second, the results of two alternative approaches to analysis will be compared: one in which exposure to the intervention is defined *a priori *in terms of place of residence (as above), the other in which exposure to the intervention is defined *post hoc *in terms of actual reported use of the guided busway [30]. This comparison will be somewhat analogous to that between an intention-to-treat analysis and a per-protocol analysis in a clinical trial. Both approaches may be viewed as a form of sensitivity analysis of the effects of the provisional decisions made in defining the 'intervention' and 'control' areas. They may reveal unexpected findings about the distances participants are prepared to walk, cycle or drive to gain access to the guided busway, thereby contributing to more accurate modelling of the transport impacts of future infrastructure projects of this kind.

### Variables and measurement instruments

#### Core questionnaire survey

Core data are collected from each participant using a questionnaire that incorporates the Recent Physical Activity Questionnaire (RPAQ). RPAQ is designed to ascertain domestic, occupational, recreational and transport-related physical activity, from which physical activity energy expenditure (PAEE) and total energy expenditure (TEE) can be estimated using an established algorithm. RPAQ is closely based on the previously-validated EPAQ2 questionnaire [39] developed for the EPIC-Norfolk cohort study and subsequently also used in intervention studies, but takes the past four weeks rather than the past year as its reference period. A validation study of RPAQ using healthy Cambridge volunteers aged 21-57 has shown the estimated PAEE to have good test-retest reliability (ICC = 0.76) and unusually strong criterion validity (r = 0.39) against PAEE objectively assessed using the doubly labelled water technique [40].

The questionnaire also includes:

• A seven-day retrospective travel record focusing on the journey to and from work, based on an instrument used (and shown to have acceptable test-retest reliability) in a previous study of active commuting [41]

• A one-day record of all journeys made on the previous day, adapted and simplified from the travel diary used in the UK National Travel Survey [42] and previously used in the M74 study in Glasgow [17]

• Items on perceived characteristics of the environment, previously used (and shown to have acceptable test-retest reliability) in the M74 study in Glasgow [17,43] but applied in this study to participants' routes to and from work [44,45] rather than to their residential environments

• Items on the mediators of changing travel mode choice predicted by the Theory of Planned Behaviour, adapted from those used in a previous intervention study and applied to car use [46]

• Items from the Self-Report Index of Habit Strength applied to car use [47]

• An item on self-reported sickness absence in the past year, previously shown to be strongly correlated with sickness absence objectively verified from employment records in the Whitehall study [48]

• The SF-8 scale for assessing general physical and mental health as a measure of wellbeing [49]

• Miscellaneous items to capture key demographic, socioeconomic and other health-related characteristics including age, gender, level of educational attainment, access to cars and bicycles, possession of a driving licence, car fuel type and engine size, presence of long-term limiting illness or disability, difficulty walking and self-reported height and weight.

The questionnaire survey is repeated annually throughout the duration of the study at the same time of year to minimise the influence of seasonal variations in travel behaviour. From 2010 onwards, the questionnaire includes items on awareness and use of the guided busway (including walking and cycling along the route, which is possible even before the busway is open to bus traffic) and reasons for using or not using the guided busway. At follow-up, participants are also asked to report any recent changes in household circumstances likely to influence travel behaviour (such as pregnancy, childbirth, children starting or moving school, or adults taking on new responsibilities at work or as carers).

#### Basic activity monitoring

A random sample of participants who indicate their willingness in principle to undertake basic activity monitoring are selected to receive an Actigraph activity monitor as well as a questionnaire in their initial survey pack. Because of the limited pool of monitors available, participants in the 'intervention' and 'control' groups are prioritised for receipt of monitors over those in the 'reserve' group. The Actigraph is a small, lightweight accelerometer that provides detailed information about the intensity, frequency and duration of physical activity, has been extensively validated in both laboratory and free-living conditions [50], and has been successfully used with high response and completion rates in previous population-based observational studies [51]. Monitors are drawn from a pool of Actigraph GT1M and GT3X models, both set to record activity counts in five-second epochs in the biaxial mode. Participants are asked to wear their monitor on an elastic waistband on the right hip during waking hours for seven days and to record on a log sheet the times at which the monitor was removed and reattached and the reasons for removal. Those who complete activity monitoring are offered simple individualised feedback on their activity data in the form of a bar chart comparing their recorded daily minutes of moderate-to-vigorous physical activity (MVPA) to the average for their age/sex group and to current public health recommendations. They are then asked to repeat the activity monitoring with their annual follow-up questionnaire surveys in order to provide longitudinal within-subject objective physical activity data.

#### Enhanced activity monitoring studies

In the year following their initial ('baseline') survey, members of the study cohort are invited to opt in to additional in-depth studies. These provide for more detailed characterisation of differences both within and between the 'intervention' and 'control' groups after adjustment for baseline covariates.

##### Household travel diaries

All follow-up participants are invited to complete a seven-day household travel diary based closely on that used in the UK National Travel Survey [42] in addition to the core questionnaire survey. The index case (main participant) in each household is asked to complete the diary day by day during the seven day monitoring period with, or on behalf of, all members of their household. The purpose of the household travel diaries is:

• To enable exploration of the interrelationships of travel behaviour within households, such as the influence of the need to take children to school en route to work, or the additional opportunity for car use by other members of the household created by a commuter's decision to cycle to work and leave the car at home

• To provide more detailed travel data from which to estimate the effects of any changes in travel behaviour on overall household travel-related carbon emissions

• To provide a seven-day travel diary for the index case (main participant) in each household for comparison with contemporaneous objective measurements where these are collected (see below).

##### Enhanced objective measurement

All members of the basic activity monitoring subgroup (those who complete Actigraph monitoring at baseline) are invited to opt in to enhanced objective measurement at follow-up. Instead of simply repeating seven days of Actigraph monitoring as at baseline, they are invited to undertake seven days of monitoring using both Actiheart combined heart rate and movement sensors and QStarz BT-Q1000X global positioning system (GPS) receivers. The accelerometer component of the Actiheart provides the equivalent of the follow-up Actigraph data for these participants [52]. Both Actiheart sensors and GPS receivers have been successfully used in previous free-living population studies [53,54]. The Actiheart is a lightweight waterproof device that clips onto two standard electrocardiogram (ECG) electrodes on the chest and measures acceleration, heart rate, heart rate variability and ECG amplitude. It has been shown to be a valid and reliable tool for measuring both acceleration and heart rate and therefore offers a more accurate assessment of PAEE than accelerometry alone, particularly for activities such as cycling which are not optimally ascertained using hip-worn accelerometers [52]. Individual calibration is not required because a simple calibration protocol based on sleeping heart rate and gender has been shown to be adequate for free-living studies [55]. Those who complete Actiheart data collection are offered more detailed individualised feedback on their activity data. The QStarz BT-Q1000X GPS receiver records the spatial coordinates (latitude and longitude) of participants at five-second intervals. Participants are asked to wear their GPS receivers during waking hours and recharge them each night - a simple procedure with which participants in previous studies have shown a high degree of compliance [54].

The purpose of enhanced objective measurement is to maximise the precision of measurement of travel behaviour and estimation of PAEE in a subgroup of participants, thereby providing robust objectively-measured data from which to:

• Determine the (combinations of) travel modes used by participants

• Estimate the PAEE involved in using different (combinations of) modes of transport

• Test the hypothesis of activity substitution between domains (e.g. that an increase in active travel may be compensated for by a decrease in leisure-time physical activity)

• Estimate the contribution of active travel to overall PAEE and thereby estimate the effect of the intervention on overall PAEE

• Identify the physical characteristics of the routes used by participants to travel to and from work and thereby examine associations between route and other environmental characteristics and choice of travel mode.

#### Longitudinal qualitative interview study

##### Semi-structured interviews

A purposive sample of participants (n~50) are invited to take part in a qualitative interview at baseline. These interviews are designed to explore the diverse practices and experiences of men and women, people in different age groups, people with and without access to a car, and people living in different areas. Interviews are conducted in participants' homes, workplaces or other convenient locations at the participants' choice and are semi-structured using a flexibly-applied topic guide. Each interview begins with an identification of the origin and destination of the participant's usual commuting journey, the usual route followed, the (combination of) mode(s) of transport usually used, and variations on the usual journey. The researcher then explores the reasons for these choices and variations (such as the need to transport children), the availability of alternatives, and what factors influence the choice between these options. Finally, the researcher explores whether participants have any expectation or intention of changing their travel mode choices, the barriers to and facilitators of making such changes, and their views as to why other people may have made other choices. Each interview is recorded using a digital voice recorder and subsequently transcribed verbatim. Participants who complete an interview at baseline may be invited to take part in a follow-up interview later in the study, along with other participants purposively selected on the basis of their responses to the core questionnaire survey.

##### Photo-elicitation interviews

A subset of the interview sample (n~20) is invited to take part in a follow-up interview several months later. After being given guidance on the rights and responsibilities associated with taking photographs [56], participants are asked to take photographs of either their actual or their ideal commuting journey using disposable cameras [57-59] or their own digital cameras. The subsequent interview is mainly guided by participants' discussion of these photographs which serve as self-generating prompts. Photo-elicitation is widely used as a research tool in qualitative health research [57,58,60] to add a participatory element and also to facilitate in-depth interviewing. In this study, it also enables the project to capture potential seasonal and other changes in travel behaviour. Finally, participant-produced photographs form visual data of the physical and social contexts in which travel takes place that can be analysed in their own right. Participants may choose to take photographs as a family or household project or in collaboration with work colleagues, in which case other household members or work colleagues may be present during the photo-elicitation interviews and informally contribute to the interview conversation [61]. Some photo interviews are also combined with a 'map-drawing' exercise for further elicitation purposes and to locate photos or interview narratives along travel routes.

##### Further ethnographic data

Interviews and photo-elicitation interviews are complemented by detailed ethnographic field notes written by the researcher, during and immediately after each interview. Field notes document the context in which interview data is produced, record other informal interactions and gather data on the environmental and social surroundings of participants' homes and journeys. Ethnographic data also include general observation of travel practices in the local area and local public discourses around travel, in order to further contextualise participants' representations of travel behaviour and social and environmental factors.

### Analysis

#### Quantitative outcome measures

The study was originally designed on the basis of a primary outcome measure defined in terms of the net change in time spent walking or cycling on the journey to and from work (daily active commuting time) after one year (min/day). In addition, the study was originally designed to examine the following secondary outcome measures:

1. Net change in active commuting time after two years (min/day)

2. Net change in total active travel time after one and two years (min/day)

3. Net change in overall physical activity expenditure estimated from self-reported data after one and two years (metabolic equivalent (MET)-h/week based on RPAQ data)

4. Net change in overall physical activity estimated from objective measurement data after one and two years (mean counts/min, and min/week spent in MVPA)

5. Net change in wellbeing after one and two years (changes in SF-8 physical and mental health summary scores)

6. Net change in self-reported sickness absence after one and two years (days/year).

Net change is defined as the average within-subject change in a given outcome measure in the 'intervention' group minus the average within-subject change in the 'control' group after adjustment for demographic, socioeconomic, geographical, psychosocial and health correlates of travel behaviour at baseline using multivariate regression analysis.

Change in these outcome measures will be studied over a two-year follow-up period in the majority of the cohort, but owing to the delayed implementation of the intervention it is likely that the greatest impact (if any) of the busway on these outcome measures will occur between the second and third years of data collection, and not between the first and second years as originally intended.

#### Sample size estimation

Data obtained in the validation study for RPAQ [40] suggest mean daily active commuting times in Cambridge of about eight minutes per day for men and five for women; various studies suggest that the standard deviation of the daily quantity of walking or active travel is likely to be of the order of 1.5 times the mean value [17,20,40,62]; and systematic reviews suggest a realistic mean increase in active travel time attributable to an effective intervention of the order of 15 to 30 min/week [22,24]. We therefore aimed to achieve a sample size of 394 in each group at follow-up (788 in total), which would provide 80% power to detect a standardised mean difference (*d*) between the intervention and control groups of 0.20 using a two-sample t-test (α = 0.05, two-sided). This equates to a mean increase in active commuting time of 2 min/day, assuming a standard deviation of 10 min/day. Such an effect size would, appropriately, be smaller than the pooled estimate reported in the Cochrane review of controlled trials to promote physical activity (*d *= 0.28 [7]) but would still be large enough to be worth detecting.

#### Quantitative analysis

Questionnaire and objective measurement data will be checked and cleaned using a combination of range and consistency checks and data cleaning algorithms and software already developed for these instruments. Actigraph, Actiheart and GPS data will also be merged and imported into a geographical information system (GIS). Network distances will be computed from the centroid of the unit postcode for each participant's home address to their nearest bus stop, their nearest bus stop on the guided busway, and their workplace. GPS time and positional data will be used to identify journey start and finish times, estimate velocities and predict the travel mode(s) used on journeys using a published protocol [63], validated against the heart rate data obtained using Actiheart, and used to estimate the proportion of PAEE attributable to commuting. Adjacent GPS data points will be joined so that routes are depicted as linear features whose surroundings can be characterised using a range of indicators such as predominant land use type and 'greenness' based on detailed land use maps and an enhanced transport network layer using a protocol developed previously [64].

The main cross-sectional analyses will consist of multivariate regression analyses of the demographic, socioeconomic, geographical, psychosocial and health-related correlates of travel mode choice and physical activity and a comparison of how the findings of that analysis differ according to which summary measures of the behaviours are used. The main longitudinal quantitative analyses will consist of multivariate regression analyses to examine the effect of access to the busway and other new transport infrastructure on changes in travel behaviour after adjustment for demographic, socioeconomic, geographical, psychosocial and health-related correlates of travel behaviour at baseline; to examine how the effect of exposure to environmental modification varies according to demographic, socioeconomic and geographical characteristics and baseline level of physical activity; and to examine the relationships between changes in travel behaviour and changes in overall physical activity, body mass index, wellbeing and sickness absence. Household travel diary data will be examined to explore relationships between the travel modes of household members using multivariate regression analysis.

#### Estimation of carbon emissions

Baseline carbon footprints (kg or tonnes CO_2_/person/day) will be calculated from survey data based on the distance, frequency, and urban or rural character of commuting journeys computed using the GIS along with data on travel mode choice, car fuel type and engine size. They will then be recalculated from follow-up survey data to quantify the overall impact of the intervention on carbon emissions attributable to travel. Household travel diary data will also be used to examine in more detail the wider knock-on effects on carbon emissions attributable to travel by other household members.

#### Criterion validation of alternative methods of ascertaining active travel

A small group of participants will complete synchronous core questionnaires, household travel diaries and enhanced objective measurement during follow-up data collection. Data from this overlap group will be used to validate the estimates of active travel obtained using questionnaires, travel diaries and accelerometers against the criterion of active travel ascertained from combined heart rate and movement sensor and GPS data. The purpose will be to inform the optimal choice of measurement instruments for future studies of this kind, which necessarily involves a trade-off between the validity, feasibility, acceptability and cost of the various instruments available ranging from questionnaires (relatively cheap and acceptable to participants, but of uncertain validity) to combined heart rate and movement sensors (relatively expensive and somewhat less convenient for participants).

#### Qualitative analysis

The analysis of the qualitative data will follow an inductive approach in order to arrive at a general, multilayered ethnographic account. Content analysis of interview transcripts (first checked against the audio recordings) and photographs (stored digitally) will be conducted by a subset of the research team to ensure reliability. By identifying thematic perceptions, experiences and practices of travel, resulting hypotheses concerning the interactions with the social worlds in which they take place will be tested by re-examining the entire qualitative dataset. Guided by social theory, the analysis will be extended beyond what is merely said and shown to address why travel, journeys and places are represented by participants in the way they are and how are these are embedded in larger societal contexts [65].

## Discussion

Few studies have set out to test the hypothesis that infrastructural improvements primarily concerned with public transport may directly or indirectly promote active travel, or to estimate the magnitude of changes in overall physical activity or other health-related impacts that may be attributable to interventions of this kind. Addressing the unresolved research and policy questions in this area is not straightforward, and the experience of designing and initiating this study illustrates several of the challenges facing researchers working on similar problems in applied public health research.

One challenge is that of effectively combining different disciplinary perspectives on the research problems. This study involves a collaboration between health, environmental and transport researchers, a combination of quantitative and qualitative approaches to gathering and analysing data, and the examination of a range of possible public health impacts [66], notably on overall physical activity and carbon emissions. These outcomes are closely related to obesity and climate change - two of the most important contemporary public health challenges which are likely to benefit from interdisciplinary research and intersectoral approaches to policymaking [67]. However, the development of common methodological ground in both measurement and evaluation in this field is still at a relatively early stage [19]. There is also considerable scope for developing new approaches to integrating quantitative and qualitative data and analyses in understanding the contexts, mechanisms and effects of interventions of this kind. The development of such 'mixed method' approaches to research will be the subject of a further paper.

A second challenge is that of implementing robust quantitative measurement of travel and physical activity behaviour in the context of an unpredictable and frequently changing intervention timetable which is completely outside the researchers' control [68]. Few previous studies of new transport infrastructure have included any measure of physical activity, let alone the combination of self-reported and objective measures used in this study [13,22,26]. The challenges of rapidly recruiting a sample and collecting baseline objective physical activity data before the scheduled opening of the new transport infrastructure will also be the subject of a further paper.

A third challenge is that of analysing a cohort dataset with repeated and varying measures of both exposures and outcomes. The busway is being constructed in a shifting political, fiscal and operational climate, and it forms only one element of a continually evolving local transport infrastructure. The study has therefore been designed as a cohort study within which it will be possible to examine the influence of environmental changes (including, but not limited to, those embodied in the busway itself) on patterns of behaviour using quasi-experimental methods within the cohort dataset. This flexible approach to study design may be essential in complex and unpredictable intervention situations of this kind, but it poses undoubted challenges for analysis: not only do different participants take part in different combinations of survey waves and optional additional measurements, but they are also exposed to different 'doses' and types of environmental change in different time periods. Although methods exist for analysing observational datasets of this kind, they have less often been applied to the evaluation of interventions. This study will therefore contribute to the development and demonstration of a growing body of methods for understanding the influence of the physical environment, and changes in the environment, on travel behaviour, physical activity and wider health impacts.

## Competing interests

The authors declare that they have no competing interests.

## Authors' contributions

DO (principal investigator) had the original idea for the study and led the design of the study and the writing of the original protocol, the applications for grant funding and ethical approval, and this manuscript. SG, AJ and RM (co-investigators) contributed to the design and the writing of the original protocol and grant application. CG devised the photo-elicitation element of the study. JP and LY contributed to the design of parts of the study through their initial work on the collection or analysis of baseline data and JP prepared Figure 1. NJ devised the spatial sampling strategy for the study and conducted an initial set of qualitative interviews that informed the design of the main series of interviews. SC contributed to the design of the qualitative elements of the study. CC (study coordinator) contributed to the design, protocol and applications for ethical approval for parts of the study. All authors contributed to the critical revision of the paper and approved the final version.

## Pre-publication history

The pre-publication history for this paper can be accessed here:

http://www.biomedcentral.com/1471-2458/10/703/prepub
